# Investigation of the Shear Thickening Fluid Encapsulation in an Orifice Coagulation Bath

**DOI:** 10.3390/polym11030519

**Published:** 2019-03-19

**Authors:** Xing Liu, Jun-Li Huo, Ting-Ting Li, Hao-Kai Peng, Jia-Horng Lin, Ching-Wen Lou

**Affiliations:** 1 Innovation Platform of Intelligent and Energy-Saving Textiles, School of Textile Science and Engineering, Tianjin Polytechnic University, Tianjin 300387, China; xliu56ncsu2019@163.com (X.L.); 13512059270@163.com (J.-L.H.); litingting_85@163.com (T.-T.L.); skyphk@163.com (H.-K.P.); 2 Tianjin and Ministry of Education Key Laboratory for Advanced Textile Composite Materials, Tianjin Polytechnic University, Tianjin 300387, China; 3 Laboratory of Fiber Application and Manufacturing, Department of Fiber and Composite Materials, Feng Chia University, Taichung 40724, Taiwan; 4 Fujian Key Laboratory of Novel Functional Textile Fibers and Materials, Minjiang University, Fuzhou 350108, China; 5 College of Textile and Clothing, Qingdao University, Qingdao 266071, China; 6 Department of Fashion Design, Asia University, Taichung 41354, Taiwan; 7 School of Chinese Medicine, China Medical University, Taichung 40402, Taiwan; 8 Department of Medical Research, China Medical University Hospital, China Medical University, Taichung 40402, Taiwan; 9 Department of Bioinformatics and Medical Engineering, Asia University, Taichung 41354, Taiwan

**Keywords:** shear thickening fluid (STF), W/O/W multiple emulsion, the orifice coagulation bath method, encapsulation

## Abstract

The orifice coagulation bath method is proposed to encapsulate shear thickening fluid (STF) to form STF capsules, in an attempt to improve the combination of STF and the matrix as well as strengthen the flexibility and stability of the STF composites. By varying the calcium chloride concentration (10, 20 mg/mL), sodium alginate concentration (5, 7, 10 mg/mL) and the surfactant dosage (10%, 20%, 30%), optimal preparation conditions were studied, considering the capsule strength and encapsulation rate. The capsules were also characterized using a scanning electron microscope (SEM), Fourier transform infrared spectroscopy (FTIR) and a thermogravimetric analyzer (TGA). The results show that the optimal solution for the preparation of the capsules is composed of 30% surfactant, 10 mg/mL mass concentration of CaCl_2_, and 10 mg/mL mass concentration of sodium alginate. The rough surface and porous interior was observed by SEM. The average diameter of the capsules was 1.93 mm. The TGA curves indicate an improvement on the capsule thermal stability. This study thus provides a promising STF capsule preparation method.

## 1. Introduction

Shear thickening fluid (STF) is a non-Newtonian fluid with a viscosity that increases with the shear rate, especially at a critical shear rate, which triggers an upsurge of viscosity [1,2,3]. STF features a unique thickening performance in dynamic mechanical properties, and thus is commonly applied in damping systems [4,5]. Via the combination of the STF, the composites show a greater resistance to vibration and damage [6,7,8,9]. As for medical devices, STF has commonly been used in the sports protective devices for joint parts, like shoulders, knees, elbows, ankles and hips, preventing damage to the joints caused by acceleration [10].

There is a popular application for STF in the design of protection materials. Klaus et al. conducted an experiment in which carbon fiber-reinforced composites were filled with STF for better durability to low-velocity transverse impact loading, and the improved resistance and energy absorption were examined [11]. Majumdar et al. immersed 3D woven aramid fabrics in STF, forming flexible body armor with strengthened stab resistance [12]. Blending foam material with STF to produce foaming composites with stronger damping properties has also attracted considerable interest [13,14,15,16]. To improve the compatibility of STF and matrices, and thus the stability and flexibility of the STF composites, the encapsulation of STF strategy was proposed and proven to be an effective resolution [17].

STF encapsulation means to encapsulate STF in the spherical membrane, after which the membrane solidifies to a shell and thus forms a solid capsule. This solidification of the membrane can be attained via chemical, physical or physicochemical methods. In particular, the orifice method, which is categorized as a physicochemical method, contributes to easy production. In addition, the capsules injected from the nozzle undergo the crosslinking-curing process and form capsules at sizes of over 500 μm. Wu et al. employed a coagulation bath to produce microcapsules of kleber’s oxytocin Rs-5 as microbial fertilizer, which increased the survival rate of bacteria when salinity stress was high [18]. Sugiuraet al. invented a new micro-fluid device, with which they produced calcium alginate microspheres about 50–200 μm with a narrow size distribution [19]. Blandino et al. used a coagulation bath to seal the biocatalystin calcium alginate gel. For further applications, the substrate and biocatalyst were in appropriate contact, because the biocatalyst was dissolved inside the capsule’s core. Calcium alginate gel was proven to have the advantages of biocompatibility, low production cost, and ease of industrialization [20]. Zhang et al. successfully encapsulated STF, obtaining double-shell STF capsules. Compared to the quasi-static compression, STF capsules exhibited an absorption nominal strain which was 154 times greater, providing an efficient shear thickening effect against dynamic impact [17].

With respect to these advantages, the orifice coagulation bath method is proposed to produce the gel beads with post-encapsulated curing. The liquid paraffin and an oil-in-water (O/W) emulsifier (Span20) were used to form the interior sphere surface, while sodium alginate and a water-in-oil (W/O) emulsifier (Span80) were made into the exterior sphere surface in order to encapsulate the STF. The mixtures were dropped through an orifice into a calcium chloride solution. Alginate and calcium chloride were cross-linked and formed the hard shell. The capsules were rinsed using deionized water and dried for the STF capsules. The liquid core (i.e., STF) of STF capsules provide damping properties when the capsules are rendered with an impact force. Moreover, the exterior calcium alginate shell can also absorb the impact energy, thus strengthening STF capsules. As a result, STF capsules have a diversity of applications, due to their damping properties regarding the mechanical force. The method we used was easier than that of the previous study in terms of preparation [17]. This study proposes a new method to encapsulate STF and will serve as a valuable reference.

## 2. Experimental

All the materials and reagents were purchased from Tianjin Sanjiang (Serida) technology, tianjin, China.

### 2.1. Preparation of Shear Thickening Fluid (STF)

40 mL of Polyethylene glycol (PEG)200 (MW 190-210) was stirred in a beaker at 100 rpm, after which 10 g silica (500 nm, ≥99.5% and MW60.08) and 80 mL of anhydrous ethanol (≥99.7%) were infused. The blends were mixed with a magnetic bar at 600 rpm for 6 h. Next, the blends were processed with ultrasonic oscillation for 3 h and then put into a vacuum in an oven for 24 h at room temperature in order to remove the air bubbles, after which it yielded STF, after the anhydrous ethanol became completely volatile.

A malvern rotating rheometer was used to test rheological properties of the resulting STF. The flat plates (pp40) with a diameter of 4 cm were fitted 3 mm apart. The oscillation rate range was 0.1–1000 rpm, while the temperature was set at 25 °C.

### 2.2. Manufacture of Capsules

**Figure 1:**
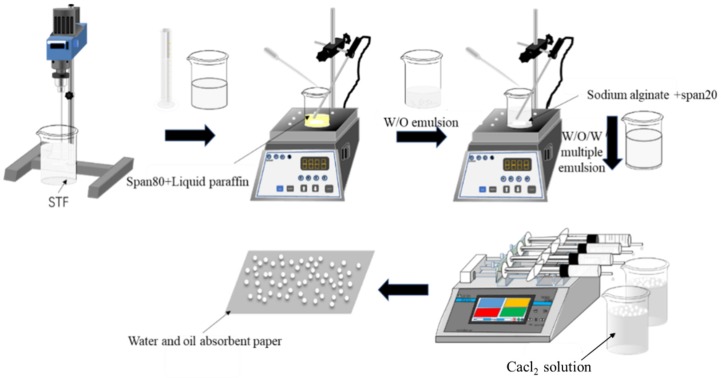
Schematic manufacturing procedure of shear thickening fluid (STF) capsules.

**Figure 2:**
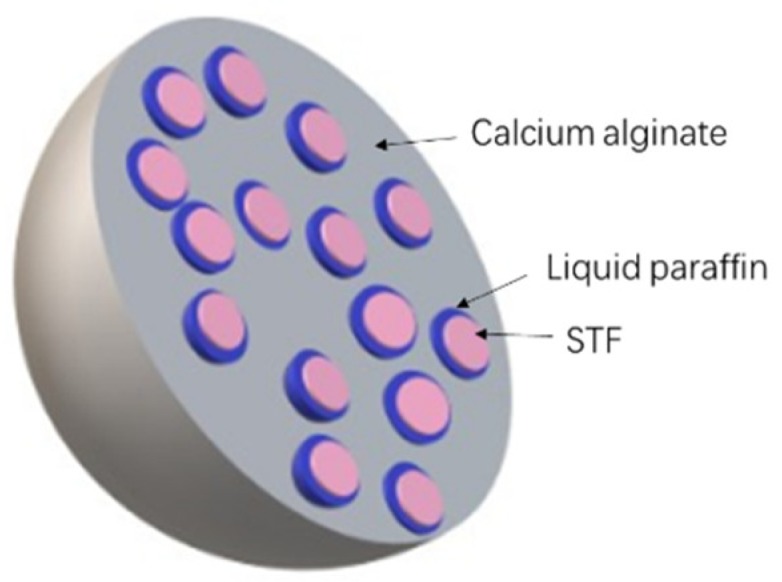
Schematic cross-section of STF capsule.

Figure 1 shows the schematic manufacturing procedure of the STF capsules. Hydrophobic surfactant (Span80, MW428.61) was added to liquid paraffin (density of 0.835–0.855) and fully mixed to prepare the STF. Next, the STF was dropped into liquid paraffin to form the W/O emulsion, followed by 30 min of stirring with a magnetic stirrer at 25 °C with a stirring speed of 1000 rpm. The second hydrophilic surfactant (Span20, MW346.46) was added to a sodium alginate solution (90%) and fully mixed, after which the W/O emulsion was slowly added. The blends were mixed for another 20 min at 25 °C with a stirring speed of 100 rpm using a magnetic stirrer, which formed the W/O/W multiple emulsion. The W/O/W multiple emulsion was sucked into a 10 mL syringe with a specified orifice diameter of 1.55 mm. The syringe was set to a microinjection pump, which operates at a driving speed of 10 μL/min, pushing the W/O/W multiple emulsion into a prepared calcium chloride solution to form spherical droplets. Subsequently, the crosslinking between sodium alginate and calcium chloride (≥96%) provided the STF capsule with a solid shell. At last, the collected capsules were rinsed with deionized water three times and dried on a water/oil absorbent paper at room temperature. The calcium-alginate shells of STF capsules were formed as a result of cross-linking sodium alginate with calcium chloride. Figure 2 shows the schematic cross-section of an STF capsule.

### 2.3. Characterization of the STF Capsule

In order to determine the optimum experimental parameters, during the experimental process the morphology of both the W/O emulsion and the W/O/W multiple emulsion were observed using an optical microscope (SAGA SG50-2A43, Suzhou, China) STF capsules are required to bear certain load without rupture during storage and processing and thus have a diversity of applications. To get strong enough capsules, various preliminary experiments have been done. In this study, the strength of the capsules was measured using an SF series Digital Force Gauge (SF-5 Wenzhou, China), examining the required force that renders compression rupture to a single capsule. During the test of capsule strength, a single capsule was fixed on the double-sided adhesive tape to ensure stability during the test, and the compression strength was recorded when the capsule was slowly pressed down at a constant speed until the rupture. Fifty samples for each specification were used for the test. According to those test results, the optimal influential factors in encapsulation regarding the strength of the capsule have been discussed.

The surface and internal structure of the capsules also affect the strength and encapsulation rate of STF capsules. The surface and cross-section of STF capsules were observed using a scanning electron microscope (SEM, TM3030, HITACHI, Tokyo, Japan). Electron microscopy samples were prepared by sticking capsules on conductive adhesive and then spraying gold for testing. A Nikon smz-10a stereomicroscope was used to measure and observe the capsule size.

**Figure 3:**
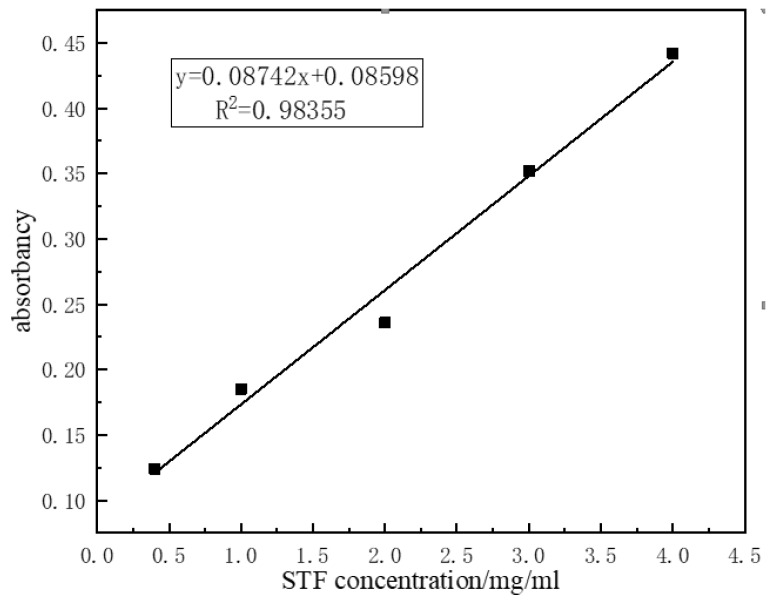
Standard curve of STF.

An ultraviolet spectrophotometer (UV2600, TECHCOMP, Shanghai, China) was used to examine the encapsulation rate by measuring the content of silica. The reference solution (i.e., control group) was PEG 200. An ultraviolet visible spectrophotometer was used to measure the absorbance, which was plotted into a curve based on the differences in wavelength. The maximum absorption wavelength is the characteristic absorption wavelength of SiO_2_ [21]. An ultraviolet spectrophotometer was used to measure the absorbance of STF and PEG, confirming that SiO_2_ has an absorption wavelength of 237 nm. Afterwards, the absorbance of STFs with concentrations of 0.4, 1, 2, 3, and 4 mg/mL were measured at 237 nm, and the test results are listed in Table 1 and then drawn in Figure 3.

The linear equation after fitting was Y = 0.08742x + 0.08598 with a correlation coefficient > 0.98. This indicates that the degree of STF concentration and absorbance fits closely with a linear correlation. Thus, the absorbance of encapsulated STF was computed using a linear equation to predict the solution concentration and the encapsulation rate.

(1) $$\mathrm{Encapsulation}\;\mathrm{rate}\;=\frac{\mathrm{Weight}\;\mathrm{of}\;{\mathrm{SiO}}_{2}\;\mathrm{in}\;\mathrm{capsule}}{\mathrm{Weight}\;\mathrm{of}\;{\mathrm{SiO}}_{2}\;\mathrm{initially}}\times 100\%$$

In order to examine whether STF was successfully encapsulated, the STF, W/O/W multiple emulsion and shells were tested for Fourier transform infrared (FTIR) (Thermo Fisher Scientific, Waltham, MA, USA) measurement using a thermo FTIR spectrometer. A smart diamond, a wavelength range of 400–4000 cm^−1^, and a resolution of 4 cm^−1^ were used.

A thermogravimeter (TG 209F3, NETZSCH, Bavaria, Germany) was used to measure the thermal stability of the capsules. The temperature scan ramped up from 40 to 600 °C at 10 °C /min.

## 3. Results and Discussion

### 3.1. Optimization of Manufacturing Parameters

#### 3.1.1. Rheological Property of Shear Thickening Fluid

The change in viscosity with corresponding shear rate can be explained by several mechanisms, especially order-disorder theory. Below the critical shear rate, STF particles have a hierarchical arrangement, which appeared to have a relatively lower viscosity. When it exceeded the critical shear rate, the shear force strengthened the hydrodynamic force on particles and disoriented the hierarchical direction, due to which the hierarchical particles transformed from being in order to disorder, which in turn caused a drastic increase in the suspension viscosity [22]. Another explanation is that the silica particles are distributed evenly and randomly in a liquid medium at the equilibrium state. With the increase in the shear rate, it transformed into a hierarchical structure, which lead to a decreased viscosity. When exceeding the critical shear rate, the stratified structure was destroyed and became clusters, which was demonstrated by the rapid increase in the viscosity, the shear thickening [23]. Egmond et al. explained that the shear thickening was related to the formation of the intermolecular structure, which was induced by shear force [24].

**Figure 4:**
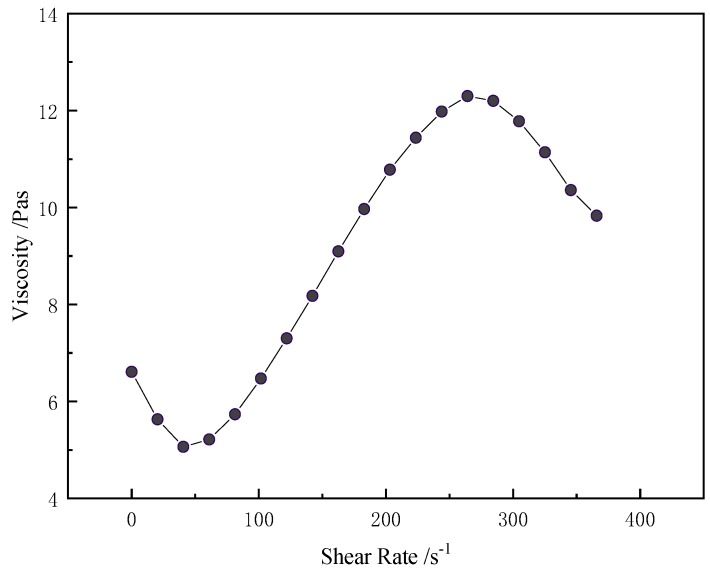
The rheological behavior of 35%STF.

Figure 4 shows the rheological properties of STF used in this study, made of the 500 nm silica and with a mass fraction of 35%. Increasing the shear rate caused the viscosity of STF to fluctuate like a lying “S”. The initial viscosity is 6.6 Pa·s and the viscosity decreased to 5.1 Pa·s at a critical shear rate of 40.7 s^−1^. The increase in the shear rate rendered a drastic upsurge of STF with a viscosity of 12.3 Pa·s, which was a response to the shear thickening behavior.

#### 3.1.2. Effect of Calcium Chloride Concentration on Performance of Capsules

**Figure 5:**
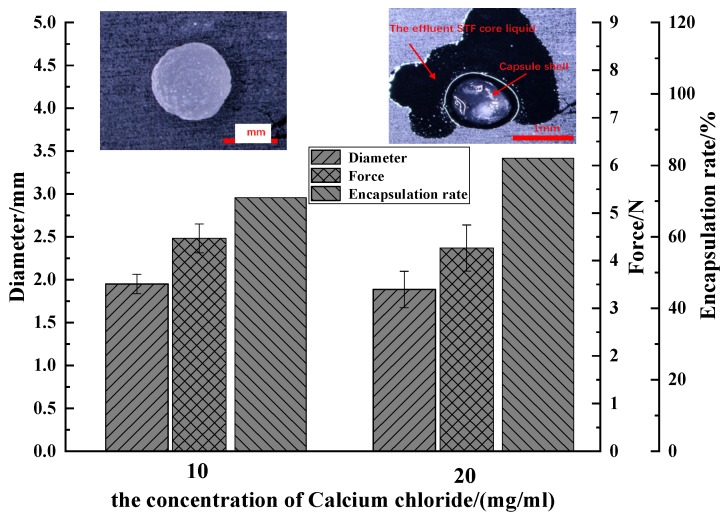
Comparison of the diameter distribution, strength and encapsulation rate of the capsules as related to the concentration of calcium chloride.

Two concentrations of the calcium chloride, 10 and 20 mg/mL, are set to be discussed. The strength and diameter of the capsules were tested, examining the effect of calcium chloride concentration. The strength of a single capsule was tested using a digital force gauge (Wenzhou, China). Fifty capsules were tested for each specification (i.e., calcium chloride concentration being 10 and 20 mg/mL). The W/O surfactant was 30%; sodium alginate mass concentration was 10 mg/mL; the proportion of W/O emulsion to sodium alginate was 1:4, the multiple emulsion stirring time was 40 min; and the liquid level spacing was 100 mm. Figure 5 demonstrates the effect of calcium chloride concentration on the capsule diameter, strength, and encapsulation rate of STF capsules.

The capsules and the breakage of capsules were observed by the stereomicroscope. The outflow of the core materials (STF) was seen as one piece of evidence of successful encapsulation. The average diameter of capsules with corresponding calcium chloride concentration was similar, 1.95 mm for 10 mg/mL and 1.88 mm for 20 mg/mL, respectively. The strength of a single capsule with corresponding calcium chloride concentration was also similar, 4.47 N for 10 mg/mL and 4.26 N for 20 mg/mL. The obvious differences can be seen for the encapsulation rate with corresponding calcium chloride concentration, 71% for 10 mg/mL and 82% for 20 mg/mL. It has been reported that, during the formation of calcium alginate microspheres, the divalent metal ions (Ca^2+^) function like a connective bridge to bound with two calcium alginate chains [25,26,27]. So, an increase in the Ca^2+^ concentration is responsible for more compact, thicker, and stronger alginate shells. However, in our experience [25], the shell thickness and strength are not separately affected by the Ca^2+^ concentration; it always involves other producing parameters.

#### 3.1.3. Effect of Sodium Alginate Concentration on the Properties of the Capsules

**Figure 6:**
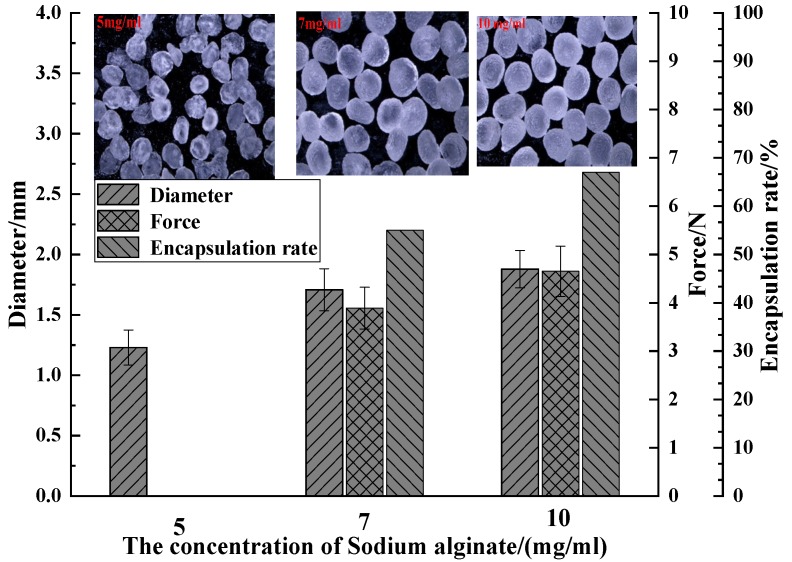
Comparison of capsule diameter distribution, strength and encapsulation rate as related to the concentration of sodium alginate solution.

Sodium alginate concentrations, 5, 7, and 10 mg/mL, were compared. The W/O surfactant was 30%; the ratio of core material to the mass of sodium alginate was 1:4, the multiple emulsion stirring time was 40 min; the calcium chloride concentration was 10 mg/mL; and the liquid level spacing was 100 mm. The strength of a single capsule was tested by the same method as mentioned before. Figure 6 shows the effect of sodium alginate concentration on the capsule diameter, strength, encapsulation rate of capsules.

The capsule diameter distribution with corresponding sodium alginate concentration is 1.23 mm for 5 mg/mL, 1.71 mm for 7 mg/mL, and 1.87 mm for 10 mg/mL. For capsules made of 5 mg/mL sodium alginate, most of them appear in a bowl shape without a liquid core and the rest are too weak, thus they were excluded from the strength test. The strength of the capsule with corresponding sodium alginate concentration was 3.89 N for 7 mg/mL and 4.65 N for 10 mg/mL. The encapsulation rate with corresponding sodium alginate concentration was 55%, and 65% for 7 and 10 mg/mL, respectively. A higher sodium alginate concentration contributes to a higher viscosity of solution, which results in a longer time for the droplets to fall, and thus an increase in the droplet diameter as well as the diameter of capsules [26]. It can also provide more binding sites for Ca^2+^ ions to build a more compact crosslinking gel structure. The compact structure strengthens the capsules in terms of force resistance. Simultaneously, compact shells also facilitate the encapsulation of multiple emulsions, preventing the drainage of core fluid and increasing the encapsulation rate.

#### 3.1.4. Effect of Surfactant on Multiple Emulsion

**Figure 7:**
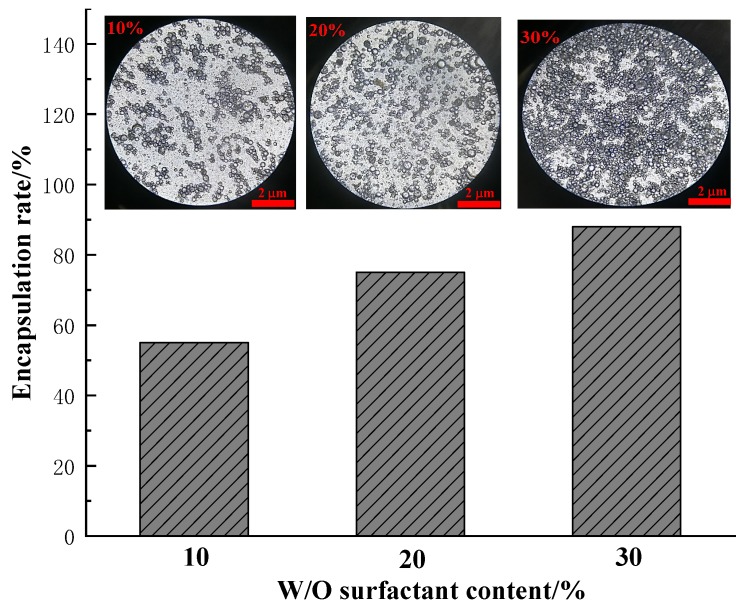
Encapsulation rate as related to W/O surfactant loading.

The effect of the surfactant, Span80, has been discussed with varying concentrations of 10%, 20% and 30% under the following conditions: a sodium alginate concentration of 10 mg/mL; a calcium chloride concentration of 10 mg/mL; and a mass ratio of core material to the sodium alginate of 1:4. The resulting capsules are compared in Figure 7.

Figure 7 shows the capsule morphology and encapsulation rate of W/O emulsion as related to different contents of surfactant. The contents of surfactant (i.e., Span 80) in W/O emulsion had an obvious impact on the encapsulation configuration of W/O emulsion, which is in agreement with other reported works [28,29,30]. The number of emulsion droplets was proportional to the addition of Span80. The higher the number of the W/O droplets, the more water phase material was enclosed. Hence, the amount of Span80 also contributes to the encapsulation ratio, which was 56%, 75%, and 88% for 10%, 20% and 30% of surfactant loadings, respectively. Moreover, an increase in surfactant strengthened the tension of the surface membrane of the droplets, which helped the capsules to encapsulate the liquid core.

### 3.2. Characterization of STF Capsules

Based on the preliminary discussion of the producing conditions mentioned above, the capsules were made with 30% surfactant, 10 mg/mL of CaCl_2_ and 10 mg/mL of sodium alginate and evaluated.

#### 3.2.1. SEM observation and Capsule Size Distribution

**Figure 8:**
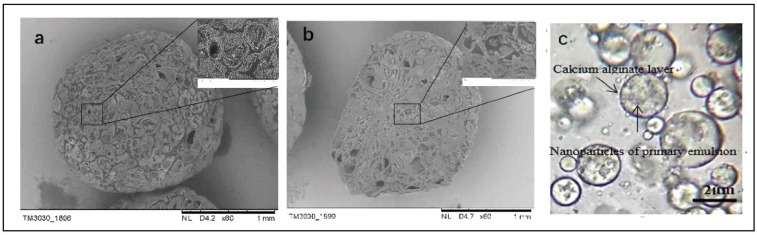
Scanning electron microscope (SEM) images of surface morphology (**a**) capsule, (**b**) the cross-section of capsule, and (**c**) optical microscope image of W/O/W multiple emulsion.

Figure 8 shows the SEM images of the surface and cross-section morphology of capsules. Figure 8a shows the rough surface and the round shape of a capsule. The capsule can be seen as the compaction of the deformed sub-capsules, with a relatively clear edge from the inset. The sub-capsules were sodium-alginate-coated liquid paraffin and STF, which is a product of the crosslinking between sodium alginate and calcium chloride solution.

Figure 8b shows the SEM image of the cross-section of capsules where there are many holes inside the capsule. It indicates that the capsules are composed of numerous solidified multi-emulsion droplets, which can also be seen in the inset. During the formation of multiple emulsions, the W/O primary emulsion enclosed with a nanoparticle and the calcium alginate outer layer were observed by an optical microscope and shown in Figure 8c.

**Figure 9:**
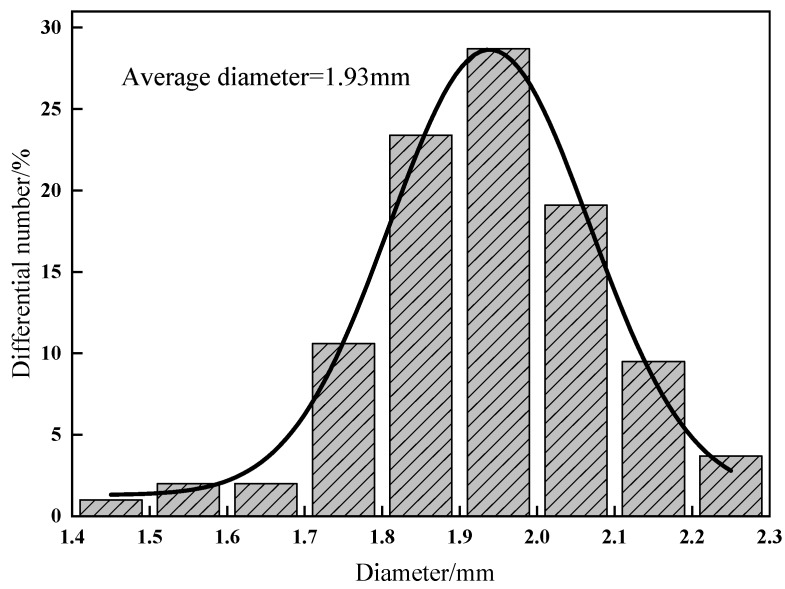
Histogram of capsule size distribution.

The capsule size distribution in Figure 9 shows a relative narrow size distribution, with the average diameter being 1.93 mm. The majority of capsules had a size of 1.8–2.1 mm, which is ascribed to the diameter of the syringe (1.55 mm). This is common for capsules produced by the orifice methods. The droplets of the multiple emulsion expand being extruded from the needle, which in turn increased the droplet size. Then, the drops were cross-linked with a calcium chloride solution and retained the same droplet size, forming the capsules.

#### 3.2.2. FTIR

**Figure 10:**
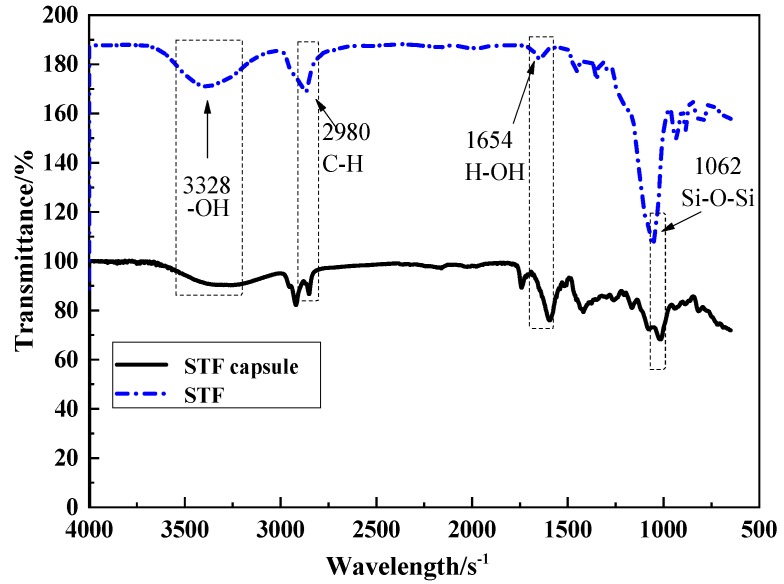
Fourier transform infrared spectroscopy (FTIR) spectra of STF capsule, W/O/W multiple emulsion and STF.

Figure 10 shows the infrared spectrum of STF and one of the typical STF capsule. The strong and wide peak at 3328 cm^−1^ is the anti-scaling vibration peak of –OH. Moreover, we can observe the presence of the double bond peak of C=C at 1603 cm^−1^, bending vibration peak of H–OH at 1654 cm^−1^, anti-scaling vibration peak Si–O–Si around 1062 cm^−1^,bending vibration absorption peak of Si-OH at 932 cm^−1^, and symmetric stretching vibration peak of Si–O at both 480 cm^−1^ and 814 cm^−1^ (i.e., characteristic peaks of silica) [27], the stretch vibration absorption peak of C–H at 2980 cm^−1^, bending vibration absorption peak of C–H at 1424 and 1359 cm^−1^ (i.e., characteristic peaks of paraffin), and the overlap symmetric stretching vibration of S–O from SiO_2_ and stretching vibration peak stack of C–O–C from PEG at 1152 cm^−1^, which are consistent with the findings of Ren et al. [28]. This indicates that STF is inside the STF capsules, or rather STF is successfully encapsulated.

#### 3.2.3. Results of Thermogravimetric Analysis of Capsule

**Figure 11:**
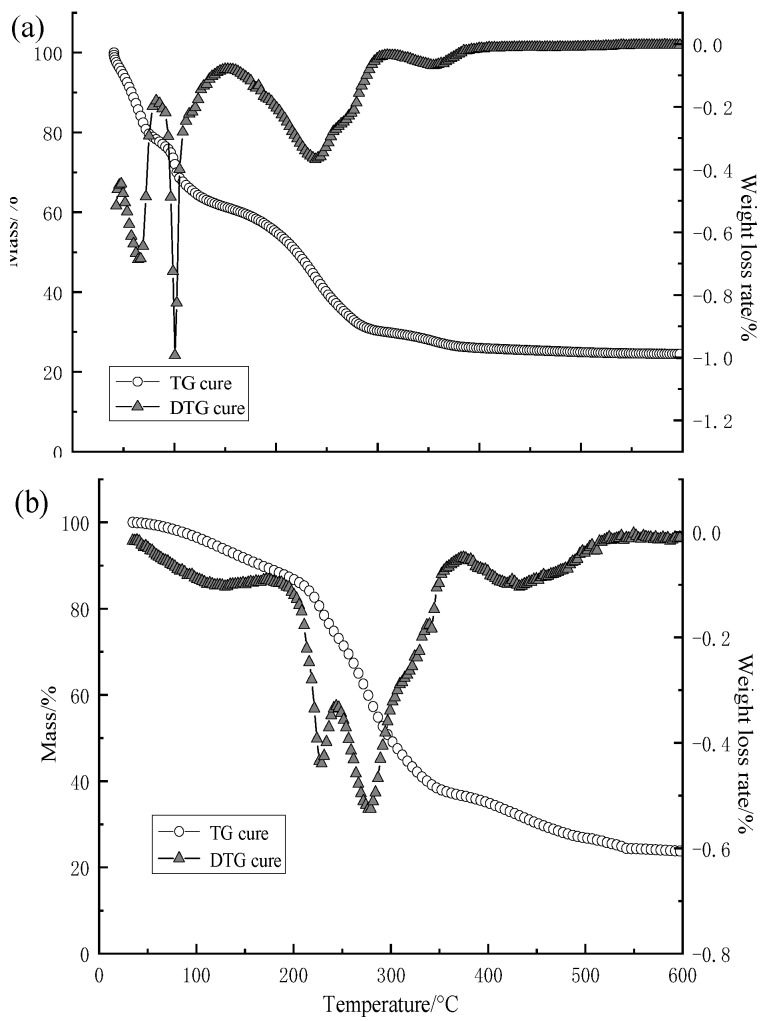

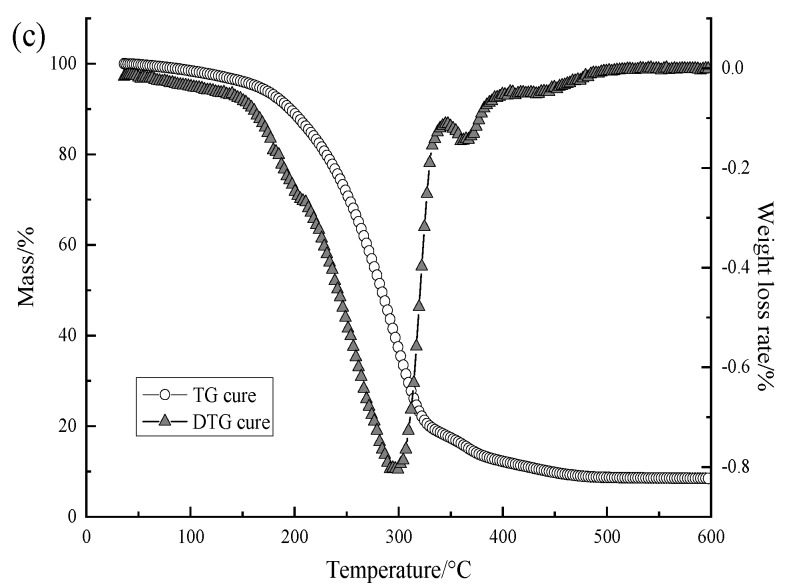
TG analysis of (**a**) W/O/W multiple emulsion; (**b**) calcium alginate shell; and (**c**) STF capsule.

Thermogravimetric analysis (TGA) is effective to characterize the thermal stability of the capsule [29,30,31]. It is also a supplementary line of evidence for the successful encapsulation. Figure 11 shows the TG analysis of STF capsules, calcium alginate shells, and W/O/W multiple emulsion.

The thermal properties of W/O/W multiple emulsion are shown in Figure 11a. Up to around 150 °C, the weight loss is rapid, and complicated refers to the evaporation of the liquid components such as PEG and water. The weight loss in the next step from 152–309 °C is attributed to the decomposition of PEG in the STF, which occurs at 270 °C. The last step of the weight loss is ascribed to the decomposition of sodium alginate around 309–394 °C, which is consistent with Figure 11b. The obvious gradual decrease from the certain protection of the emulsion outer layer indicates the core-shell structure.

The TA curves of the capsule calcium alginate shell can be divided into four stages, which occur at 0–184 °C, 184–243 °C, 243–377 °C, and 377–522 °C, respectively, in Figure 11b. When capsules are heated, the water in the shells starts to evaporate. With a specified temperature of 184 °C, the weight rate drastically increases, which suggests that the chains of calcium alginate start to break, and its decomposition can be divided into two stages. The first stage involves an inflection point at 226 °C where the decomposition of span20 occurs. Moreover, the second stage involves an inflection point at 276 °C where the decomposition of sodium alginate occurs (*T*_d_ = 285 °C). At the stage of 377–522 °C with an inflection point of 429 °C, it could be ascribed to the decomposition of calcium alginate.

The TG and DTG curves in Figure 11c show the two stages of the decomposition of capsules, which occur at 200–347 °C and 347–389 °C. The initial mass loss of the capsule in the first stage is 80%, which is ascribed to a gradual evaporation of the liquid core inside the capsule. In addition, the weight loss in the second stage is 8.93%, which is attributed to the shell decomposition. The interior of capsules appears porous and the share of shell is low. By comparing the TG curves of W/O/W emulsion and the STF capsules from Figure 11a,c, it is obvious that the thermal properties of the capsule are better than those of the emulsion. It is also a piece of supplementary evidence for the encapsulation.

## 4. Conclusions

In this study, we presented an orifice coagulation bath method to produce an STF capsule that is composed of 35% 500 nm STF as the core, liquid paraffin as the interior shell, and calcium alginate as the exterior shell. The optimal parameters were chosen to be 10 mg/mL CaCl_2_, 10 mg/mL sodium and 30% W/O surfactant. SEM observation showed the rough and porous surface and the honeycomb interior of the capsule, which suggest that the internal structure of STF capsules is suitable for applications in the absorption of mechanical energy. The capsule size distribution was normally distributed, with an average size of 1.93 mm. FTIR and the TG results show that STF was successfully sealed in the capsules. TG analyses also show that STF capsules demonstrate a good thermal stability when the temperature is lower than 226.3 °C. Comparison of the TGA curves of the liquid core and shells of capsules indicate that the combination of the shells efficiently improved the thermal stability of STF capsules.

## Figures and Tables

**Table 1:** Relation between concentration of silica and absorbance of STF.

SiO_2_ Concentration/(mg/mL)	0.4	1	2	3	4
Absorbance	0.124	0.197	0.226	0.352	0.442
